# Interleukin-17A Mediates Acquired Immunity to Pneumococcal Colonization

**DOI:** 10.1371/journal.ppat.1000159

**Published:** 2008-09-19

**Authors:** Ying-Jie Lu, Jane Gross, Debby Bogaert, Adam Finn, Linda Bagrade, Qibo Zhang, Jay K. Kolls, Amit Srivastava, Anna Lundgren, Sophie Forte, Claudette M. Thompson, Kathleen F. Harney, Porter W. Anderson, Marc Lipsitch, Richard Malley

**Affiliations:** 1 Division of Infectious Diseases, Department of Medicine, Children's Hospital, and Harvard Medical School, Boston, Massachusetts, United States of America; 2 Division of Pulmonary Medicine, Department of Medicine, Children's Hospital, and Harvard Medical School, Boston, Massachusetts, United States of America; 3 Departments of Epidemiology and Immunology and Infectious Diseases, Harvard School of Public Health, Boston, Massachusetts, United States of America; 4 Departments of Clinical Sciences at South Bristol, Academic Unit of Child Health, and of Cellular and Molecular Medicine, University of Bristol, Bristol, United Kingdom; 5 Division of Pulmonology, Department of Pediatrics, Children's Hospital of Pittsburgh and the University of Pittsburgh, Pittsburgh, Pennsylvania, United States of America; 6 Göteborg University Vaccine Research Institute (GUVAX), Department of Microbiology and Immunology, Institute of Biomedicine, Göteborg University, Sweden; 7 Department of Obstetrics and Gynecology, Cambridge Health Alliance, Cambridge, Massachusetts, United States of America; University of Toronto, Canada

## Abstract

Although anticapsular antibodies confer serotype-specific immunity to pneumococci, children increase their ability to clear colonization before these antibodies appear, suggesting involvement of other mechanisms. We previously reported that intranasal immunization of mice with pneumococci confers CD4+ T cell–dependent, antibody- and serotype-independent protection against colonization. Here we show that this immunity, rather than preventing initiation of carriage, accelerates clearance over several days, accompanied by neutrophilic infiltration of the nasopharyngeal mucosa. Adoptive transfer of immune CD4+ T cells was sufficient to confer immunity to naïve RAG1^−/−^ mice. A critical role of interleukin (IL)-17A was demonstrated: mice lacking interferon-γ or IL-4 were protected, but not mice lacking IL-17A receptor or mice with neutrophil depletion. *In vitro* expression of IL-17A in response to pneumococci was assayed: lymphoid tissue from vaccinated mice expressed significantly more IL-17A than controls, and IL-17A expression from peripheral blood samples from immunized mice predicted protection *in vivo*. IL-17A was elicited by pneumococcal stimulation of tonsillar cells of children or adult blood but not cord blood. IL-17A increased pneumococcal killing by human neutrophils both in the absence and in the presence of antibodies and complement. We conclude that IL-17A mediates pneumococcal immunity in mice and probably in humans; its elicitation *in vitro* could help in the development of candidate pneumococcal vaccines.

## Author Summary

The bacterium *Streptococcus pneumoniae* (pneumococcus) causes serious disease in children and the elderly, including pneumonia and meningitis (inflammation of the brain). Carriage of pneumococcus in the nose is a necessary first step for most infections. As children age, they carry pneumococcus for shorter periods of time and their risk of disease decreases also. The mechanisms underlying this age-related decrease of carriage are not well understood. A deeper understanding of resistance to colonization would enable us to develop better pneumococcal vaccines. Using experimental mouse models, we show that repeated exposure to pneumococci leads to a subsequent reduction in duration of pneumococcal carriage, similar to what is observed in humans. We identify the immune cells that are responsible for this process, so-called T_H_17 cells, which release a factor that enables human blood cells to kill pneumococcus more efficiently. We show that these T_H_17 cells exist in adults and children, but not in newborn babies, which suggests that they may arise as a consequence of humans being exposed to pneumococcus. We describe an assay for the measurement of these cells in humans. Such an assay could facilitate the development of novel vaccines directed against pneumococcal carriage.

## Introduction

*Streptococcus pneumoniae* (pneumococcus) is an “extracellular” pathogen, generally considered to be killed by phagocytic ingestion, which is facilitated by opsonic antibodies. The success of anti-pneumococcal serum therapy using passive transfer of serotype-specific antibodies [1] and of vaccinations based on purified or conjugated capsular antigens [2],[3] clearly shows that anticapsular antibodies protect humans against pneumococcal colonization and disease. There is good epidemiologic evidence for the importance of such immunity in certain common serotypes [4],[5]. However, we and others have found that factors other than anticapsular antibodies may play a role in the natural development of protection against pneumococcal colonization and disease. First, the reduction in pneumococcal disease incidence after the first year of life occurs simultaneously for both rare and common serotypes, suggesting the acquisition of one rather than many individual immune responses [6]. Similarly, the duration of carriage of many serotypes declines steeply between the first and second birthdays for many serotypes [7]. Since experience with conjugate vaccines has suggested that anticapsular antibodies reduce incidence of carriage but leave duration unaffected [8], this observation also suggests a mechanism of acquired immunity other than anticapsular antibodies. Moreover, the declines in carriage duration and invasive disease incidence precede by several years the detection of naturally-acquired anticapsular antibody in most children [6],[7]. Experimental [9],[10] and observational [4],[11] studies in adults have found little or no evidence that higher anticapsular antibody concentrations are associated with greater protection from colonization. Pneumococci also express non-capsular antigens common among serotypes, and certain of these have been found to elicit antibodies with protective potential in animal models. The role of such antibodies in human immunity has been evaluated [12],[13],[14],[15],[16],[17].

Surprisingly however, recent studies have shown that immunity in mice to pneumococcal colonization acquired from prior exposure to live bacteria [18] or a killed, whole-cell vaccine [WCV, consisting of killed pneumococcal whole cell antigen (WCA) with cholera toxin (CT) as an adjuvant] [19] is independent of antibodies of any specificity, and clearance of longstanding carriage in previously unexposed animals can likewise be antibody-independent [20]. Immunity had been shown to be dependent on the presence of CD4+ T cells at the time of challenge [18],[19], but the co-participation of specific immune factors other than antibody was not ruled out.

Here we show that intranasal immunization with the WCV confers protection against experimental pneumococcal colonization via the chemoattractant and neutrophil activating cytokine IL-17A, in a neutrophil-dependent fashion. Methods were devised to assay expression of IL-17A *in vitro* using peripheral blood samples. IL-17A expression by peripheral blood of WCV-immunized mice is highly correlated with subsequent protection against colonization, and expression by human cells, including those from adults and children, can be shown as well. Finally, we developed a surface phagocytosis assay with which we show that IL-17A enhances pneumococcal killing by human polymorphonuclear cells in the absence as well as presence of opsonins.

The data indicate the possibility that IL-17A responses play a role in naturally-acquired immunity to pneumococcus in humans and that assay of this cytokine *in vitro* may assist in the evaluation of certain candidate pneumococcal vaccines that target mucosal colonization.

## Results

### Prior exposure of mice to killed or live pneumococci reduces the duration of experimental pneumococcal carriage

**Figure 1:**
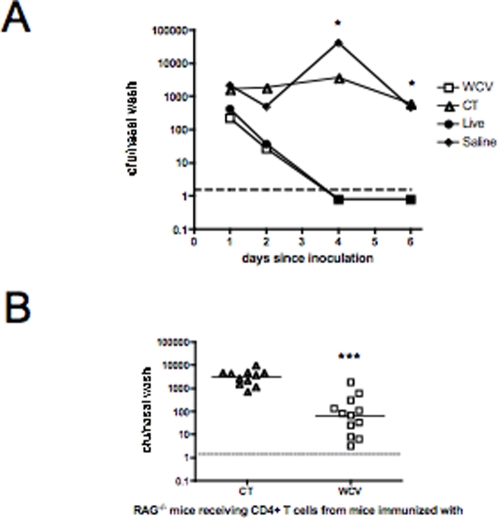
Duration of carriage and effect of adoptive transfer following immunization with killed or live pneumococci. A. Effect of intranasal immunization with WCV or live exposure upon density of pneumococcal colonization in C57BL/6 mice. Density of colonization in mice immunized with WCV vs. CT alone or repeatedly exposed to live pneumococcal strain 0603 vs. saline alone at various time points (n = 4 per time point) following challenge. By day 4, both the incidence and density of carriage were significantly lower in mice immunized with WCV or exposed to live pneumococcus compared to mice immunized with CT or saline, respectively. * P<0.05. The dashed line represents the lower limit of detection of bacterial colonization. B. Effect of adoptive transfer of CD4+ T cells from WCV-immunized mice on pneumococcal colonization of recipient, unimmunized RAG1^−/−^ mice. Each data point represents the density of nasopharyngeal colonization in cfu/ nasal wash for each mouse one week post-challenge. The horizontal bar shows the geometric mean cfu/nasal wash for each group and the dashed line represents the lower limit of detection of bacterial colonization. C57BL/6 mice were immunized with WCV or CT as indicated. Four weeks after the last immunization, CD4+ T cells were harvested from splenocytes of mice and infused into naïve, unimmunized RAG1^−/−^ mice; challenge of these mice and quantification of colonization was then performed. RAG1^−/−^ mice that received CD4+ T cells from WCV-immunized mice had significantly lower density of colonization than mice that received cells from CT-immunized mice (*** P<0.0001 by Mann-Whitney U).

The duration of carriage was followed after intranasal challenge with serotype 6B pneumococci 4 weeks post-exposure to WCV. Both WCV-vaccinated and control mice immunized with CT alone were colonized one day after challenge. In mice immunized with WCV however, carriage became significantly reduced after 4 days compared to controls given cholera toxin (CT) adjuvant alone (median density of colonization on day 4 in WCV- vs. CT-immunized mice 251 vs. 3720 cfu/nasal wash, P = 0.029 by Mann-Whitney U test) and was undetectable by day 6 (0/4 WCV-immunized mice had detectable colonies on day 6 vs. 4/4 mice that received CT, P = 0.029 by Fisher's Exact test, Figure 1A). A similar differential was observed in mice that had been repeatedly exposed to live pneumococci vs. saline controls: the density of colonization became significantly different by day 4 after inoculation (Figure 1A). By day 6, similar to what we observed in WCV-immunized mice, 0/4 mice exposed to live pneumococci had detectable colonies compared to 4/4 saline controls (P = 0.029 by Fisher's exact test). When data from all time points were compared, mice immunized with WCV or exposed to live pneumococci had a significantly shorter time to clearance compared to their respective CT or saline controls (P = 0.0001 for comparison of WCV vs. CT and P = 0.004 for comparison of live exposure vs. saline). Thus the protection by prior pneumococcal exposure involves not immediate blockage of colonization but rather an accelerated clearance over days. Subsequent studies compared WCV-vaccinated with control animals 7 days after the intranasal challenge.

### CD4+ T cells transfer acquired immunity to pneumococcal colonization to RAG1^−/−^ mice

Several previous studies showed that acquired immunity to pneumococcal colonization in mice can be antibody-independent and dependent on CD4+ T cells [19],[20],[21],[22]. Here, adoptive transfer showed that CD4+ T cells are not only necessary but also sufficient for the accelerated clearance induced by WCV: unimmunized Rag1^−/−^ mice (lacking both B and T cells) were infused with CD4+ T cells from mice immunized with WCV or CT alone. The RAG1^−/−^ mice that received CD4+ T cells from WCV-immunized wild-type mice had significantly reduced density of colonization by day 7 compared to mice infused with CD4+ T cells from mice immunized with CT alone (P = 0.0001 by Mann-Whitney U, Figure 1B).

### Acquired immunity to pneumococcal colonization is associated with the T_H_17 subset of CD4+ T cells

**Figure 2:**
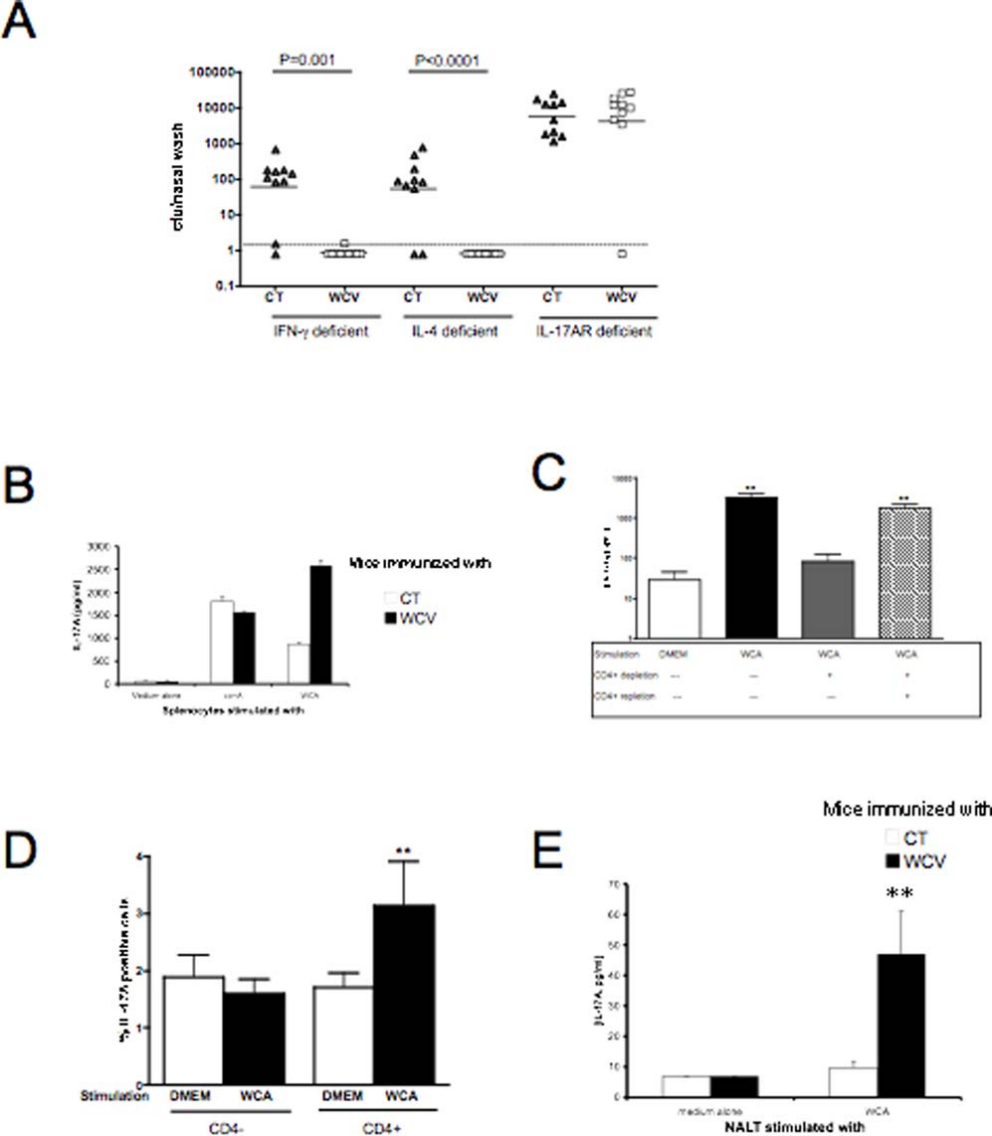
Role of T-helper-subset-associated cytokines in protection from nasopharyngeal colonization. A. Mice defective in IFN-γ, IL-4 or IL-17A receptor were immunized as described, then challenged with pneumococcal strain 0603. Mice with IFN-γ or IL-4 deficiency were significantly protected by WCV (P<0.001 vs. respective CT controls) whereas IL-17A receptor deficient mice were not protected (P>0.5 vs. CT). Dashed line represents the lower limit of detection of bacterial colonization. B. Expression of IL-17A from splenocytes of WCV-immunized mice. Cultured splenocytes from mice immunized with WCV (black columns) or CT alone (white columns) were stimulated for 72 hours with medium alone, Concanavalin A (5 µg/ml), WCA (10 µg dry weight) after which IL-17A production was measured by ELISA. Significantly more IL-17A was expressed following WCA stimulation of WCV-immunized vs. CT-immunized mice, although response to concanavalin A was similar. C. Effect of CD4+ T cell depletion upon IL-17A expression from splenocytes. Splenocytes (without or with CD4+ T cell depletion) from mice immunized with WCV were stimulated for 72 hours with medium alone or WCA after which IL-17A was measured by ELISA. IL-17A expression in splenocytes following WCA stimulation was significantly higher in the presence of CD4+ T cells compared to stimulation with medium alone or when CD4+ T cells were depleted. Repletion of CD4+ T cells restored the response. ** P<0.01 compared to cells stimulated with medium alone. D. IL-17A intracellular staining of splenocytes from WCV immunized mice. Splenocytes from WCV immunized mice were stimulated with WCA, blocked with monensin, harvested and stained for CD4+ and intracellular IL-17A as described. There is a statistically significant increase in CD4+ IL-17A positive cells following stimulation with WCA, which is not observed in the CD4- population. No increase in IL-17A positive cells could be detected in cells from unimmunized mice (data not shown). **P = 0.008 for comparison of frequency of IL-17A-positive cells in absence and presence of WCA stimulation among CD4+ cells. Data shown here are representative of three experiments, including at least 5 mice per experiment. E. Expression of IL-17A from NALT of WCV- vs. CT-immunized mice. Cultured splenocytes from mice immunized with WCV (black columns) or CT alone (white columns) were stimulated for 72 hours with medium alone or with WCA (10 µg dry weight) after which IL-17A production was measured by ELISA. Significantly more IL-17A was expressed following WCA stimulation of WCV-immunized vs. CT-immunized mice. **P<0.01 for comparison of IL-17A in WCV vs. CT-immunized mice following stimulation with WCA.

To evaluate which CD4+ T cell subset is responsible for protection, IFN-γ, IL-4 or IL-17A receptor (IL-17AR) knockout mice were immunized with WCV vs. CT alone. IFN- γ- and IL-4-deficient mice immunized with WCV were significantly protected against colonization both with respect to proportion of colonized mice (P<0.001 by Fisher's Exact test for comparison of % of colonization in WCV- and CT-immunized IFN-γ- or IL-4-deficient mice) and density of colonization (P≤0.001 compared to their respective CT controls, Figure 2A). In contrast, mice with a targeted deletion of the IL-17A receptor were not protected (P>0.5 vs. CT controls for % colonized mice or density of colonization, Figure 2A). It is noteworthy that IL-17AR-knockout mice in the CT control group had, on average, a ten-fold greater density of colonization than the corresponding IFN-γ or IL-4 deficient mice, suggesting that IL-17A may also be involved in resistance to colonization in naïve mice.

Splenocytes from mice immunized with WCV expressed significantly more IL-17A in response to WCA *in vitro* than cells from CT control animals (Figure 2B). We have previously shown that immunization with WCV confers protection against NP colonization in a CD4+ T cell dependent fashion [19]; additional experiments were performed here to confirm that IL-17A is produced by CD4+ T cells. Depletion of CD4+ T cells effectively abrogated IL-17A expression from WCA-stimulated splenocytes, such that there was no detectable increase in IL-17A production compared to splenocytes stimulated with medium alone; repletion of CD4+ T cells restored the IL-17A response seen with nondepleted splenocytes stimulated with WCA (Figure 2C). Moreover, intracellular cytokine staining experiments confirmed that IL-17A production originates primarily from CD4+ cells (Figure 2D). Splenocytes from WCV-immunized animals were stimulated with WCA and analyzed by flow cytometry. The frequency of IL-17A positive cells increased 2.3 fold among CD4+ T cells (3.37% in WCA stimulated cells vs. 1.49% in cells stimulated with medium alone, P = 0.008 by Mann-Whitney U) whereas the non-CD4+ population was unaffected (1.5% vs. 1.7% for stimulation with WCA vs. medium alone in CD4- cells, P = 0.5). Similar analyses were performed using splenocytes from mice immunized with CT alone; no increase in IL-17A positive cells was noted, either in the CD4+ or CD4- population (data not shown). Collectively, these results indicate that IL-17A is produced by CD4+ T cells.

Next, we harvested nasal associated lymphoid tissue (NALT) from WCV- and CT-immunized mice. Cells were incubated for 3 days in the presence of medium alone or with WCA, after which IL-17A expression was measured by ELISA. NALT cells from WCV-immunized mice showed significantly greater IL-17A production than CT controls in response to stimulation with WCA (Figure 2E).

**Figure 3:**
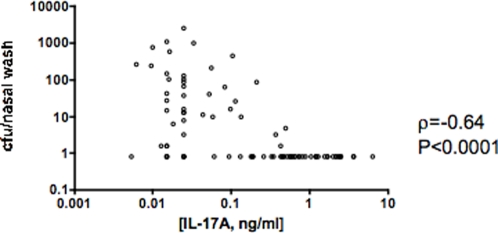
Correlation of IL-17A expression and density of nasopharyngeal colonization in mice. Three weeks after immunization of mice (n = 90) with CT with doses of WCA ranging from 1 to 100 µg, and one week before pneumococcal challenge, blood samples were obtained and stimulated with WCA (10 µg) for 6 days, after which supernatants were collected and assayed for IL-17A concentration. The correlation between density of colonization (cfu/nasal wash) 7 days after challenge and pre-challenge IL-17A expression was evaluated. IL-17A expression was significantly correlated with density of colonization.

To test whether the capacity to produce IL-17A predicted an individual mouse's protection, a total of 90 mice were intranasally immunized with CT (1 µg) plus a range of doses of WCA ranging from 1 to 100 µg and blood samples were taken 7 days before challenge and stimulated with WCA *in vitro* for IL-17A production. The IL-17A concentrations following 6 days of culture *in vitro* varied from undetectable (<0.02 ng/ml) to about 6 ng/ml, and the cfu of pneumococci/nasal wash 7 days post-challenge varied from undetectable (<1.6 cfu/nasal wash) to about 3000; there was a strong inverse correlation (Spearman ρ = −0.62, P <0.0001, Figure 3); 95% of mice with pre-challenge IL-17A concentrations above 0.3 ng/ml were free of pneumococcal colonization.

### Neutrophil-like cells are required for acquired immunity to pneumococcal carriage

Because IL-17 A induces neutrophil recruitment and activation [23], we evaluated whether neutrophils were required for protection against colonization. Mice intranasally immunized with WCV (or CT alone) were challenged, with or without administration of monoclonal antibody RB6-8C5 (which targets neutrophil-like cells) at the time of challenge. Several experiments were performed to ensure that treatment with this antibody did not affect CD4+ T cell number or function. Evaluation of splenocytes of antibody-treated animals showed no reduction in the CD4+ T cell population (data not shown). Furthermore, we confirmed that IL-17A production from the peripheral blood or from NALT of WCV-immunized mice was not affected by treatment with RB6-8C5 antibody. The peripheral blood IL-17A expression from immunized, neutrophil-depleted mice was similar to that of immunized, nondepleted mice (median IL-17A whole blood expression in neutrophil depleted vs. non-depleted mice: 1059 vs. 1290 pg/ml, P = 0.7 by Mann-Whitney U test); similarly, there was no reduction in NALT IL-17A expression from immunized mice following neutrophil depletion (median IL-17A expression from NALT in depleted vs. nondepleted mice 23.6 vs. 19.8 pg/ml, P = 0.69 by Mann-Whitney U test).

**Figure 4:**
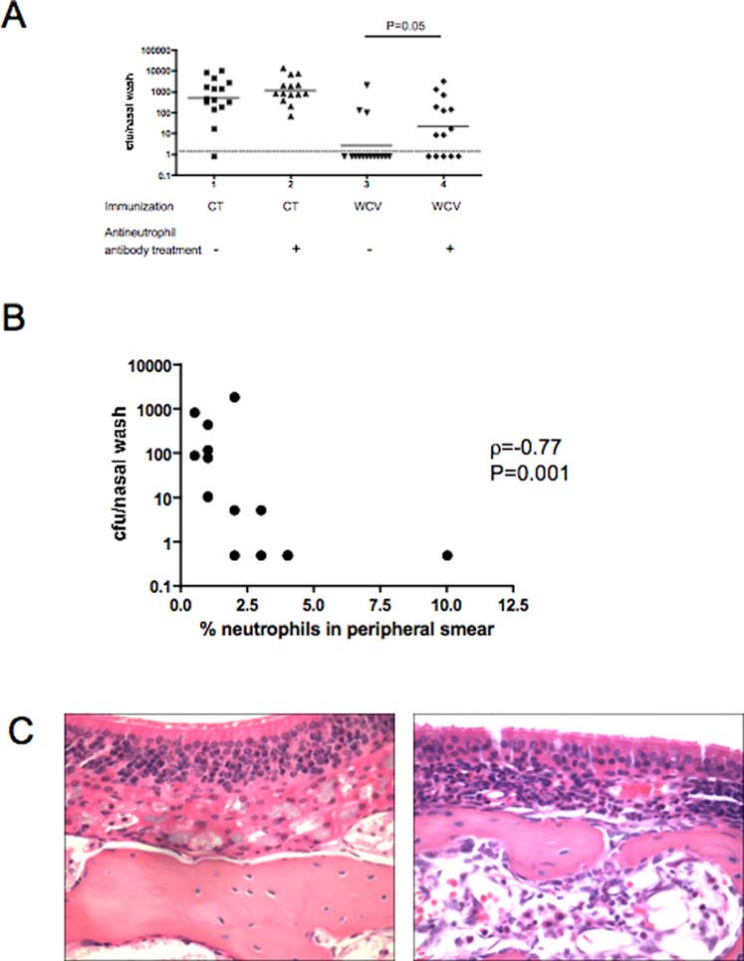
Effect of neutrophils on WCV-induced protection against pneumococcal colonization. A. Effect of neutrophil depletion on WCV-induced protection from nasopharyngeal colonization. Each data point represents the density of nasopharyngeal colonization in cfu/nasal wash for each mouse. The horizontal bar shows the geometric mean cfu/nasal wash for each group and the dashed line shows the lower limit of detection of bacterial colonization. Mice were immunized with CT or WCV as indicated; just prior to the time of challenge, mice were randomized to receive antineutrophil antibody vs. saline. Proportion of colonized mice and density of colonization was determined 7 days post challenge. WCV-immunized mice that received saline treatment were significantly better protected than WCV-immunized mice that received antineutrophil antibody, with a lower proportion of colonized mice (P = 0.025 by Fisher's Exact) and density of colonization (P = 0.05 by Mann-Whitney U). B. Correlation between neutrophil count and density of pneumococcal colonization. Neutrophil counts following neutrophil depletion were assayed at the time of sacrifice and plotted against density of colonization. There was a strong negative association between neutrophil counts and colonization density (Spearman *ρ* = −0.75). C. Histopathology of nasopharyngeal tissue following nasopharyngeal challenge of CT- (left panel) and WCV-immunized (right panel) mice. Seven days post pneumococcal challenge, mice were euthanized, heads stored in formalin, and H&E sections of nasopharyngeal tissue prepared and examined under light microscopy at 60× magnification. The presence of a dense neutrophilic infiltrate in the submucosa at the junction of the olfactory and respiratory epithelium was noted in WCV-immunized mice following pneumococcal nasopharyngeal challenge but not in CT-immunized mice. The two slides shown are representative of a total of 15 examined specimens (8 WCV-immunized and 7 CT controls, all at day 7 post pneumococcal challenge). Lesions like those represented here were observed in 6/8 immunized mice and 0/7 controls.

Neutrophil depletion significantly diminished protection by immunization (Figure 4A). WCV-immunized and neutrophil depleted mice had both higher proportion of colonized mice (9/14 vs. 3/15 colonized mice for neutrophil-depleted vs. non-depleted WCV-immunized mice respectively, P = 0.025 by Fisher's Exact test) and density of colonization (median 12.8 cfu/nasal wash vs. 0.8 cfu/nasal wash respectively, P = 0.05 by Mann-Whitney U). While WCV-immunized, neutrophil-depleted mice had reduced colonization density compared to mice that received CT alone (median colonization density 453 cfu/nasal wash, P = 0.006 by Mann-Whitney U), the percentage of remaining neutrophils was strongly negatively correlated with recovered cfu from challenged mice (Spearman ρ = −0.77, P = 0.001, Figure 4B), suggesting that residual protection was accounted for in large part by incomplete neutrophil depletion.

Consistent with these results, blinded review of histopathology of nasopharyngeal tissue of 6/8 WCV-immunized mice seven days after challenge with pneumococcus showed a distinct neutrophilic infiltrate in the submucosa at the junction of the olfactory and respiratory epithelium (Figure 4C, right panel), which is not seen in CT-immunized subsequently challenged with pneumococci (left panel) (presence of infiltrate in 6/8 WCV immunized mice vs. 0/7 CT controls, P = 0.007). Thus the data support a role for IL-17A acting upon neutrophils in protection against pneumococcal colonization in mice.

### IL-17A expression in human samples following pneumococcal stimulation

**Figure 5:**
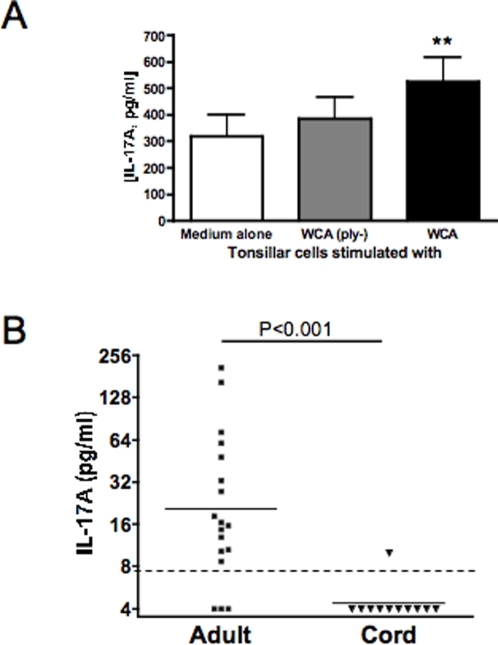
Effect of exposure to pneumococcus on IL-17A expression from human tissues and cells. A. Expression of IL-17A from tonsillar mononuclear cells from children. Tonsillar cells (n = 8) were cultured as described and stimulated with WCA or WCA derived from an isogenic, pneumolysin-negative strain (WCAply-). Stimulation with WCA was associated with significantly increased IL-17A expression compared to exposure to medium alone (P = 0.008 by Wilcoxon signed rank test), whereas stimulation with WCAply- did not increase IL-17A production. B. Expression of IL-17A from peripheral blood of adults and umbilical cord blood. Peripheral blood samples from adults (healthy adult volunteers (n = 7), parturient women (n = 11) and umbilical cord blood (n = 11) were stimulated with WCA for 6 days after which IL-17A concentration was assayed by ELISA. IL-17A production was significantly greater in adults than cord blood (P<0.001 by Mann-Whitney U test).

Next we determined whether IL-17A responses to pneumococcus could be measured in humans. Tonsillar mononuclear cells (from 8 donors) were stimulated with medium alone, WCA obtained from a pneumolysin-negative strain (WCA(ply-)) or WCA from the wild-type strain. IL-17A expression measured at 72 hours was significantly higher following stimulation with WCA than with medium alone (Fig 5A); this increase was abrogated when a pneumolysin-negative WCA was used as stimulus, consistent with prior findings in humans and in mice regarding the association between T cell-mediated responses to this toxin and prevention of pneumococcal colonization ([24] and unpublished data). Furthermore, whole blood from unimmunized adult human volunteers, presumed to have been naturally exposed to pneumococcus, produced IL-17A in response to WCA *in vitro* (Fig 5B). Eighteen subjects produced a range of IL-17A concentrations from about 4 to 200 pg/ml, with a geometric mean of 20 pg/ml. Of these volunteers, 11 were parturient women, whose geometric mean IL-17A expression was 18 pg/ml. Umbilical cord blood was available in each of these cases; IL-17A in these samples was at the lower limit of detection of the assay (4 pg/ml) in all but one case, significantly lower than that of all adult subjects or parturient women (P<0.001 and P<0.01, respectively by Mann-Whitney U).

### IL-17A enhances in vitro phagocytic killing of pneumococci

**Figure 6:**
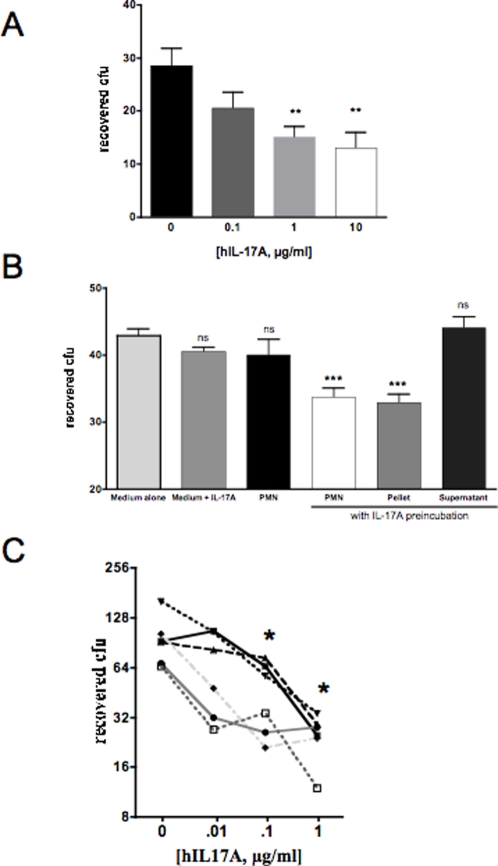
IL-17A-mediated enhanced killing of *S. pneumoniae*. A and B. Effect of human IL-17A on surface phagocytic killing of *S. pneumoniae*. A. Isolated neutrophils from healthy adult volunteers were incubated with recombinant human IL-17A at the indicated concentrations and evaluated in a surface phagocytic killing assay with pneumococcal strain 0603; colonies were counted after overnight incubation at 37°C with 5% CO_2_. IL-17A induces a dose-dependent enhancement of neutrophil killing of *S. pneumoniae* (P = 0.01 for 1 or 10 µg of IL-17A vs. no added IL-17A). B. Supernatant obtained from neutrophils after incubation with IL-17A did not have any demonstrable antipneumococcal effect, whereas washed neutrophils after incubation with IL-17A demonstrated enhanced killing. C. Effect of human IL-17A on opsonophagocytic killing of *S. pneumoniae*. Neutrophils purified from the peripheral blood of healthy adult volunteers were incubated with pneumococci anticapsular antibodies, complement, and a range of concentrations of IL-17A as indicated for 90 minutes, following which viable counts were obtained by plating on blood agar plates. Each line represents a different volunteer. IL-17A enhanced killing of pneumococci in a dose-dependent fashion in 6/6 subjects. *P = 0.016 by Wilcoxon matched pairs test.

We evaluated whether IL-17A enhances *in vitro* killing of pneumococci by human neutrophils in different assays. Having reported previously that WCV induced protection in antibody-deficient mice, we developed a surface phagocytic killing assay to evaluate whether -17A could potentiate killing of non-opsonized pneumococci. Neutrophils isolated from healthy volunteers were pre-incubated with recombinant human IL-17A, then overlaid on pneumococci that had been plated onto blood agar. The overlay of IL-17A in the absence of neutrophils did not result in any killing, consistent with studies in which the addition of IL-17A to culture medium did not affect growth of pneumococci and arguing against any direct killing effect of the cytokine or contaminant present in the preparation (as shown in Figure 6B). In the presence of neutrophils, IL-17A induced dose-dependent killing of pneumococci (Figure 6A). Thus IL-17A potentiated *in vitro* neutrophil killing of pneumococcus, in the absence of antibodies or complement.

To evaluate whether the potentiating effect of IL-17A is the result of lysis or degranulation of neutrophils, we performed trypan blue exclusion studies of neutrophils with and without preincubation with IL-17A at the highest dose studied (10 µg/ml). There was no difference in viability when IL-17A was added; over 95% of cells remained viable after 30 minutes of incubation. Furthermore, neutrophils preincubated with IL-17A then washed showed the same enhancement of pneumococcal killing as incubated neutrophils that were not washed. In contrast, the supernatant obtained after incubation of neutrophils and IL-17A had no detectable antipneumococcal activity (Figure 6B). Taken together, these data argue against a toxic or degranulating effect of IL-17A on neutrophils and are more consistent with enhancement of phagocytic activity of neutrophils by this cytokine.

Because capsular and noncapsular antipneumococcal antibodies gradually increase with age in children [6],[25],[26], we also evaluated whether IL-17A would potentiate opsonophagocytic killing of *S. pneumoniae* in the presence of limiting amounts of antibody. Bacterial polysaccharide immune globulin (BPIG) is a plasma concentrate from volunteers immunized with bacterial polysaccharides including pneumococcal serotype 6B [27]. To evaluate whether IL-17A would synergize with antipneumococcal antibodies, we added IL-17A to an opsonophagocytic assay using a suboptimal dose of BPIG. When IL-17A was added to the reaction, killing of pneumococcus was enhanced in a dose-dependent fashion in all individuals tested (Figure 6C). Killing was significantly increased when either 1 µg or 10 µg/ml IL-17A were added (P = 0.016 by Wilcoxon matched pairs test). There was no significant pneumococcal killing in the assay in the absence of any of the principal components, including PMNs, antibody (BPIG) or complement (data not shown). Additionally, as in the surface phagocytosis assay, IL-17A alone had no effect on growth of bacteria *in vitro* or survival in our assay, implying that direct killing of pneumococci by IL-17A is very unlikely.

## Discussion

The importance of CD4+ T cells in protection against pulmonary infections became clearly evident with the advent of the HIV epidemic. Infected individuals have a dramatically increased risk of infections with opportunistic pulmonary pathogens such as *Mycobacterium tuberculosis* or *Pneumocystis jiroveci* and this risk is inversely related to the number of circulating CD4+ T cells [28],[29]. For *S. pneumoniae*, HIV infection confers a 50-fold increased risk of infection, which is also inversely related to CD4+ T cell count [30],[31]. Most recently, a study in Zambian mothers has demonstrated that HIV infection is associated with a significantly increased risk of colonization and reduced time to new colonization [32]. Various hypotheses have been advanced to explain why CD4+ T cell-deficiency is associated with such a high risk of infection with pneumococcus, a primarily extracellular encapsulated bacterium; these include reduced opsonic activity of anticapsular antibodies [33], loss of memory B cells [34], and alteration of innate pulmonary immunity [35]. To date, however, it is fair to state that the paradox remains unexplained. The data presented here suggest that a loss of T_H_17 cells may also contribute to this increased susceptibility.

While the ability of pneumococcal conjugate vaccine-induced anticapsular antibodies to protect against pneumococcal colonization is clear, less is known about the natural development of immunity to pneumococcal colonization. There appear to be both antibody-dependent and antibody-independent mechanisms that reduce the likelihood or duration of carriage. Several studies have documented a homotypic anticapsular serum antibody response to colonizing pneumococcal serotypes [4],[36],[37],[38]. In a longitudinal study examining the relationship between antibodies and carriage in adults, Goldblatt et al. showed that, among six serotypes tested, anticapsular antibody concentration to serotype 14 was significantly associated with reduced odds of carriage [4]. More recently, by analyzing longitudinal carriage data from Israeli toddlers in daycare, we found a lower risk of colonization with type 6A, 14, and 23F after previous exposure to the homologous type [5]. For types 14 and 23F, this specific protection correlated with increased serotype-specific antibody concentration. On the other hand, as in the previous study [4], there was no evidence for such a correlation with several other serotypes. Several studies have argued against the role of anticapsular antibody. For example, the risk of acquisition of new pneumococcal strains in adults with chronic obstructive pulmonary disease was associated with *higher* preacquisition concentrations of anticapsular and noncapsular pneumococcal antibodies [11]. These results suggest that in this population, antipneumococcal antibodies are markers of prior exposure and perhaps *greater* susceptibility rather than predictors of protection. Finally, in the sole published example of experimental pneumococcal colonization of humans, antibodies to the capsular polysaccharide did not predict protection against colonization [39].

We, and others, have presented data supporting a role of CD4+ T cells, independently of antibody, in reducing pneumococcal colonization in mouse models [18],[19],[20],[22],[40]. In studies involving immunization with the pneumococcal zwitterionic conserved cell wall polysaccharide, we showed that neutralization of IL-17A significantly reduced protection, albeit not completely [21]. Zhang et al. then showed that reduced CD4+ T cell proliferative responses to the pneumococcal toxin pneumolysin were associated with nasopharyngeal pneumococcal carriage in children [24]. We subsequently showed that immunization with a mixture of a pneumolysin nontoxic mutant and two other pneumococcal proteins elicits T_H_17 cells and confers protection against colonization in a CD4+ T cell-dependent, antibody-independent fashion [22]. Others have argued against this possibility, proposing instead a T_H_1, IFN-γ mediated mechanism, based on the finding that IL-12p40-deficient mice cleared pneumococcal colonization as well as wild-type mice [20]. Since that report, it has become clear that IL-23, which is lacking in IL-12p40 deficient mice, is not absolutely required for the generation of T_H_17 cells as once was thought, but instead participates in their maintenance or expansion [41],[42].

Here we present evidence that acquired CD4+ T_H_17 cells reduce the duration of experimental colonization with *S. pneumoniae* in a manner reminiscent of the age-dependent decline in duration of carriage [7] and that this mechanism occurs independently of key T_H_1 or T_H_2 cytokines, IFN- γ and IL-4 respectively. We show that CD4+ T cells are sufficient to provide the protection against colonization, which is abrogated in the absence of the IL-17A receptor and highly dependent on neutrophils, one of the main targets of this cytokine. Recombinant human IL-17A enhances both antibody-independent and –dependent killing of *S. pneumoniae in vitro*. Importantly, IL-17A expression can be induced by exposure to pneumococcal antigens of tonsillar cells from children and peripheral blood from healthy adult volunteers, but not in umbilical cord blood, consistent with the view that this responsiveness may be the result of prior exposure to the pathogen.

IL-17A signaling been shown to participate in host defense against extracellular pathogens, such as *Klebsiella* and *Candida* in naïve mice [43],[44]. Prior to this report, there have been two demonstrations of a role of T_H_17 cells in vaccine-induced immunity and in both cases, whole organisms, killed or live, were used. Higgins et al. showed that protection against *Bordetella pertusssis* with a whole cell vaccine induced T_H_17-dependent protection and Khader et al. presented similar findings with the mycobacterial protein ESAT6-induced protection against *M. tuberculosis* [45],[46],[47]. The data derived from both mouse and human studies in the present report thus add to the growing evidence that T_H_17 cells contribute to immunity to respiratory pathogens.

Numerous attempts have been made to define correlates of protection against pneumococcal carriage and have focused on the humoral response to pneumococcal capsular or noncapsular antigens [4],[15],[48]; although associations between levels of antibodies in saliva and reduced risk of otitis media have been reported [48], no reliable correlate has been identified. Here we show that the IL-17A response in immunized mice is highly correlated with reduced carriage; in particular above a certain concentration, colonization beyond 7 days is very unlikely. Naturally-exposed humans have low, but measurable IL-17A responses, which could be evaluated in response to immunization with candidate pneumococcal vaccines, such as the whole cell vaccine currently under development. The demonstrated association between carriage and reduced T cell proliferative responses to pneumolysin in childhood [24] provides further support for a functional T cell assay such as the one proposed here.

## Materials and Methods

### Bacterial strains and immunogens

*S. pneumoniae* strain 0603 is a serotype 6B clinical strain [49]. Frozen mid-log phase aliquots were diluted to ∼10^6^ cfu/10 µl of intranasal inoculum for challenge. The whole cell antigen (WCA) was derived from strain Rx1AL-, a capsule- and autolysin-negative mutant and prepared as described [49]. A pneumolysin-negative WCA (WCA(ply-)) was derived from an isogenic, pneumolysin-negative strain of Rx1AL- using methods previously described [18]. The final vaccine mixture (whole cell antigen WCA + adjuvant CT) for routine immunization contained 100 µg (dry weight) of WCA plus 1 µg of CT (List Biological Laboratories, Campbell, CA) per 10 µl dose. For potency comparisons, lower amounts of WCA were used (ranging from 0.1 µg to 10 µg). For all experiments, control mice were immunized nasally with 1 µg of CT in 10 µl saline.

### Animal models

The animal model used in these experiments has been previously described [49]. C57BL/6J mice or mutants in the same background (female, age 6 weeks, Jackson Laboratories, Bar Harbor, ME) were randomized to receive 10 µl of whole cell vaccine or adjuvant alone intranasally twice at one week interval. Three weeks following the last inoculation, mice were anesthetized for retro-orbital blood sampling. One week later, mice were challenged intranasally with ∼10^6^ cfu of strain 0603. At 1 week after challenge, the mice were euthanized by CO_2_ inhalation; an upper respiratory wash was done by instilling sterile, nonbacteriostatic saline retrograde through the transected trachea and collecting the first 6 drops (about 0.1 ml) from the nostrils. Following collection of nasopharyngeal samples, the heads of WCV- and CT-immunized mice were removed and placed in formalin prior to histopathological preparation with hematoxylin and eosin (H&E) staining.

To evaluate the time to eradication of carriage, in a separate experiment, 4 mice from groups of 16 each were sacrificed at 1, 2, 4 and 6 days post inoculation and sequential dilutions of nasal washes were plated. To test whether CD4+ T cells are sufficient for protection, adoptive transfer experiments were performed. Splenocytes from wild type C57Bl/6 mice immunized with WCV or CT alone were harvested 2 months after the last immunization and CD4+ T cells were purified by magnetic bead positive selection (Miltenyi Biotec, Auburn, CA). A total of 3×10^6^ CD4+ T cells were injected retro-orbitally in naïve RAG1^−/−^ mice (B6.129S7-Rag1^tm1Mom^/J) that lack both B and T cells. The following day, these mice were challenged intranasally with strain 0603; one week later, density of colonization was determined as described above.

To determine which T cell subset is responsible for protection, mice in the C57BL/6 background and deficient in IFN-γ (B6.129S7-Ifng^tm1Ts^/J, Jackson Laboratories, Bar Harbor, ME) IL-4 (B6.129P2-Il4^tm1Cgn^/J, Jackson Laboratories) or the IL-17 receptor (B6.129 IL17Ra^−/−^ [50]) were immunized and challenged as described above. For neutrophil depletion experiments, mice were immunized as described above; on days −1, +1 and +4 relative to challenge, mice were injected intraperitoneally with 100 µg of antineutrophil monoclonal antibody RB6-8C5 (purified from myeloma cell line by Bio Express, Lebanon, NH), a rat anti-mouse IgG2b directed against Ly-6G on the surface of murine myeloid (and limited subpopulations of lymphoid) lineage cells; in pilot experiments, this regimen resulted in >90% depletion of neutrophils in most mice, although variability was observed. Because of this variability, peripheral neutrophil counts were determined at the time of euthanasia and correlated with the number of recovered pneumococci from that animal.

### Measurement of IL-17A secretion by splenocytes

Cellular suspensions of splenocytes were obtained by passing spleens from immunized or control mice through a 70-µm cell strainer (BD Biosciences, Bedford, MA). After washing and removal of red blood cells by hemolysis, cells were plated into 24-well tissue culture plates at a concentration of 5×10^6^ cells/well in 500 µl of DMEM/F12 with L-glutamine supplemented with 10% fetal calf-serum, 50 µM 2-mercaptoethanol (Sigma), and 10 µg/ml ciprofloxacin. Following 72-hour stimulation with concanavalin A (5 µg/ml, Sigma) or WCA (equivalent to 10^6^ cfu/ml), supernatants were collected following centrifugation and stored at −80°C until analyzed by ELISA for IL-17A concentration (R&D Systems, Minneapolis, MN). Supernatants were analyzed in duplicate and read against a standard, following directions provided by the manufacturer.

For CD4+ T cell depletion, splenocytes were harvested as described above. CD4+ T cells were depleted from half of each spleen by magnetic bead selection (Miltenyi Biotec, Auburn, CA) following instructions by the manufacturer. Flow cytometry confirmed removal of >95% CD4+ T cells (data not shown). Cells were seeded at the same concentration as described above (5×10^6^ cells/well). In some cases, we repleted CD4+ T cells from depleted splenocytes, by adding 10^6^ CD4+ T cells in the relevant wells.

### Intracellular staining for IL-17A

Splenocytes were harvested, seeded, and stimulated with medium or WCA (10 µg/ml) as described above. Twenty-four later, monensin (BD GolgiStop, BD Biosciences) was added as per the manufacturer's instructions and cells were harvested 12 hours later. Cells were washed, stained with anti-CD4+ antibody (antiCD4+-PE, BD Biosciences) in the presence of Fc block, permeabilized with Perm/Wash buffer (BD Biosciences), and incubated with antimouse IL17A Alexa Fluor-647 (eBioscience) for 30 minutes. Intracellular cytokine staining for IL-17A was compared in CD4- or CD4+ cells in medium alone or following stimulation with WCA. Samples were analyzed on a Cytomation MoFlo (Beckman Coulter, Fullerton, CA), and results analyzed with Summit Version 4.3 (Dako, Fort Collins, CO).

### Measurement of IL-17A secretion by NALT

NALT was harvested from immunized and control mice as described [51]. Mice were euthanized humanely, bled via intracardiac puncture to avoid blood contamination, and placed on a dissection board. The mouth was opened wide to expose the palate, which was cut carefully, so that the strips of NALT could be easily peeled off. These strips of cells were collected in medium (DMEM/F12 with L-glutamine supplemented with 10% fetal calf-serum, 50 µM 2-mercaptoethanol (Sigma), and 10 µg/ml ciprofloxacin) on ice. Cells were passed through a 70 µm strainer as described above and plated at 3×10^5^ cells/well in a 96-well tissue culture plate in a total volume of 100 µl. Cells were stimulated with medium with or without added WCA (10 µg/ml) for a total of 3 days, after which supernatants were collected and assayed for IL-17A concentration by ELISA as above.

### Measurement of IL-17A secretion by whole blood

For whole blood assays, blood of mice or humans at a final concentration of 10% was incubated in DMEM/F12 with L-glutamine supplemented with 10% fetal calf-serum, 50 µM 2-mercaptoethanol (Sigma), and 10 µg/ml ciprofloxacin in the absence or presence of killed pneumococcal antigen (corresponding to 10^7^ cfu/ml for mice and 10^6^ cfu/ml for human samples). Supernatants were collected after 6 days and the concentration of IL-17A measured as above for mice and, for human samples, by IL-17A ELISA (eBioscience Inc, San Diego, CA).

### Human subjects and samples

For peripheral blood, samples were obtained at Children's Hospital Boston (for healthy adult volunteers) or from Cambridge Health Alliance, Cambridge, MA (for parturient women or umbilical cord) after written informed consent had been obtained. The studies were approved by the Children's Hospital Boston and Cambridge Health Alliance research ethics committees. For tonsillar specimens, tonsils were obtained from children who were 2 to 12 years old (median age, 5 years), were undergoing tonsillectomy for hypertrophy, and were otherwise healthy at Bristol Royal Hospital for Children, Bristol, United Kingdom. Patients who were immunized against pneumococcus previously, who had received antibiotics within 2 weeks of the operation or steroids, or who had an immunodeficiency or serious infection were excluded. The study was approved by the South Bristol local research ethics committee and written informed consent was obtained in all cases.

### Agar surface phagocytic killing without opsonins

This assay approximates the “surface phagocytosis” described by Smith and Wood [52]. Neutrophils were isolated from heparinized blood by density gradient centrifugation (Histopaque, Sigma) following manufacturer's instructions. Neutrophils were washed extensively then resuspended in Hanks' Balanced Solution (+ Ca^2+^ and Mg^2+^) with 0.2% bovine serum albumin (Sigma), then co-incubated for 30 minutes at 37°C with recombinant human IL-17A (R&D Biosystems) at different concentrations. In some experiments, the cells were harvested by centrifugation and the supernatant collected, to examine whether the potentiating effect of IL-17A could be detected with the supernatant alone. Between 8–10 replicates of 10 µl of a bacterial suspension containing on average 100 cfu of strain 0603 were plated onto blood agar and the fluid allowed to adsorb into the agar for 15 min; 15 µl of the neutrophil suspension was overlaid and allowed to adsorb; the plates were incubated at 37°C with 5% C02 overnight after which colonies were counted.

### Phagocytic killing in suspension with suboptimal opsonization

Neutrophils were isolated from whole blood as described above, washed twice with cold Hanks Balanced Salt Solution (HBSS-) (Mediatech, Herndon, VA), and resuspended to a final concentration of 6×10^6^ cells/ml in cold HBSS containing calcium and magnesium (HBSS+) (Cellgro Mediatech, Herndon, VA) then held on ice until used. Cell counts were determined on a standard hemocytometer by counting viable cells (as determined by an absence of blue staining in the presence of Trypan Blue (Cellgro Mediatech, Herndon, VA)). *S. pneumoniae* (strain 0603 [49]) was diluted in HBSS+ to a final concentration of 5×10^4^ bacteria/ml and incubated with antibodies to pneumococcal polysaccharide (Bacterial Polysaccharide Immune Globulin, BPIG-8, a kind gift of Dr. George Siber, consisting of concentrated IgG obtained from serum of adult volunteers immunized with pneumococcal, *Haemophilus* and meningococcal polysaccharide vaccines [27]) diluted in HBSS+. The reaction was incubated at 37°C for 15 minutes rotating at 200 RPM to promote bacterial opsonization. After bacterial opsonization, the opsonophagocytic killing reaction was initiated with the addition of baby rabbit complement (Pelfreez Biologicals, Rogers, AR) and neutrophils (ratio of 1∶200 bacteria∶cells) with or without recombinant human IL-17A (R&D Systems, Minneapolis, MN) at 0.01, 0.1 or 1 µg/ml. A 1∶1600 dilution of BPIG was chosen to give sub-optimal bacterial killing (<50% killing when compared to the same conditions without BPIG) in the presence of complement and neutrophils. The opsonophagocytic killing assay was performed in a 96-well round-bottom plate (Thermo Fisher Scientific, Waltham, MA) at 37°C for 90 minutes rotating at 200 RPM. After incubation, the opsonophagocytic reaction was diluted two fold and aliquots of each reaction were plated on blood agar plates then incubated at 37°C with 5% CO2 overnight.

### Isolation and culture of tonsillar mononuclear cells

Mononuclear cells were isolated by using methods described previously [53],[54]. Tonsillar MNC were washed in sterile phosphate-buffered saline (PBS) and resuspended at a concentration of 4×10^6^ cells/ml in RPMI medium containing HEPES, 2 mM glutamine, 100 U/ml penicillin, 100 µg/ml streptomycin, and 10% fetal bovine serum (Sigma, Dorset, United Kingdom). Cells were cultured in 96-well culture plates (Corning Inc, Corning, NY), and cell culture supernatants were collected at predetermined times and stored at −70°C until assays for human IL-17A were performed by sandwich ELISA (R&D Biosystems).

### Statistical analysis

Incidence of carriage was compared by Fisher's exact test and colonization density in challenged mice was compared by the Mann-Whitney *U* test. Statistical significance of the difference between time-to-clearance curves was assessed as follows. For each group *i* (*i* = WCV, CT, live, or naïve), the proportion of mice cleared at each time point *t*, *p_i_*(*t*), was calculated. Using the max-min formula for isotonic regression [55], these proportions were smoothed to assure they were nondecreasing in *t*, yielding smoothed proportions *q_i_*(*t*). Then, a test statistic was calculated to quantify the distance between the smoothed curves for two groups (e.g., WCV vs. CT): 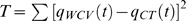. The significance level of this test statistic was estimated by permuting the group identifiers of the cleared mice at each time point, fixing the total number of mice in each group and the total number cleared at each time point. 100,000 replicates of the permuted data were obtained, and *T* was calculated for each. The p value was calculated as the fraction of these 100,000 permutations having a test statistic strictly less than that calculated for the data. The correlation between neutrophil count or IL-17A concentration and colonization density was determined by Spearman rank correlation. The effect of increasing IL-17A concentrations on enhancing killing of pneumococcus was assessed by Wilcoxon matched pairs test. For all comparisons, P<0.05 was considered to represent a significant difference.
